# Looking back at ECSS 2014

**DOI:** 10.1186/s40798-015-0018-8

**Published:** 2015-05-23

**Authors:** Steve McMillan

**Affiliations:** Springer Science + Business Media 5 The Warehouse Way, Northcote, Auckland, New Zealand

## Abstract

With the 2015 European College of Sports Science (ECSS) meeting in Malmö just around the corner (in time, if not space), I thought I would recap on what was an exciting meeting in Amsterdam last July.

As Deputy Editor of a journal that, at the time, was yet to be launched - the 19th ECSS meeting provided the perfect place to make acquaintance with new and potential Editorial Board members and to get a feel for the research landscape.

Despite the name, ECSS is an International organisation, and the delegation was truly global. Aside from Europe - Japan, Australasia and South Africa all seemed to be particularly well represented among delegates; not to mention, Aspetar, of Qatar, was a major sponsor of the event, and two exchange symposia were co-hosted between the ECSS and the American College of Sports Medicine (ACSM), and between ECSS and the Japanese Society of Physical Fitness & Sports Medicine (JSPFSM), respectively.

The 4-day conference, held at the RAI Convention Centre, consisted of 4 plenary sessions and an honorary session, 36 invited symposia, 74 other oral sessions, 106 mini-oral sessions, and over 500 E-posters, covering the full gamut of sport and exercise science. As the programme’s welcome message alluded to, I ‘regretted that I had only two ears and two eyes’; however, in saying that, the vast coverage of the conference through social media provided one with no excuse for missing out on any information.

## Sport science around the canals

As often is the case with scientific meetings, ECSS 2014 had a theme, ‘Sport Science Around the Canals’, that remarked on the wonderful venue, but really did nothing to contextualise the focus of the meeting. If there were a true theme to the meeting, I believe it was football. With the World Cup in full swing at the time, football was the most popular topic of conversation during the breaks; it also provided an icebreaker for a large number of presenters - seemingly regardless of area of research being presented.

Ahead of the conference, Aspetar hosted a satellite symposium entitled, ‘How to win the match: The science behind football’; and on the concluding afternoon, Jens Bangsbo gave the Tom Reilly Memorial Lecture, on ‘The effect of intensified training on performance and muscle adaptations in well-trained individuals’, which revolved around Jen’s extensive research in elite footballers. Finally, as enthralling as many of the presentations were, I think many delegates would agree that the culmination of the conference was when everyone came together for the live showing of Netherlands quarter final defeat of Costa Rica during the closing party at the Nemo Science Center.

Aside from football, topics that frequently arose throughout the 2014 conference included: pacing; interval training (particularly high-intensity interval training) in just about every thinkable population; physical activity in children; and lifestyle activity . . . or lack thereof (i.e. time spent sitting vs standing vs moving).

Overall, highlights included the following:

## Samuele Marcora’s brain endurance training

This, to me, seemed ground-breaking: using mentally fatiguing tasks as training stimuli to improve endurance performance. Twenty-eight healthy, physically active males were randomised to 12 weeks of standard aerobic training on a cycle ergometer either performing mentally fatiguing tasks while exercising or not. Improvements in VO_2_max were similar in both groups, but those subjected to the mental tasks experienced significantly larger improvements in time-to-exhaustion and significant reductions in ratings of perceived exhaustion, indicating that the Brain Endurance Training reduces perception of effort and improves endurance performance. Whether such training could provide elite athletes with an edge is the next question for Samuele’s research team.

## Colin Boreham’s defence for the elderly in the plenary session entitled, ‘Who has the future in public health - young or old?’

While Mai Chin A Paw began the plenary by providing a very satisfying presentation reinforcing the well-held and broadly applicable belief that ‘an investment in children is an investment in the future’; Colin Boreham argued that, actually, despite concern about physical activity levels among today’s children - in general, children under the age of 12 years remain more physically active than any other population. Meanwhile, the chronic diseases and frailty associated with old age mean that the real healthcare burden is with the growing elderly population. While even low training loads have been shown to provide tangible health benefits in the elderly, there is currently very little evidence to suggest that physical exercise behaviours promoted to children are carried through to adulthood or whether they have any positive effect on health outcomes in the long term. Colin noted the aging population has been described as the second biggest problem facing developed nations after climate change. He concluded that, given limited healthcare resources, ‘the promotion of exercise in children seems a luxury that must take second place to the elderly’.

While I, personally, found this argument to be quite convincing, it stirred a lot of debate during question time.

## David Mann’s tracking of the visual-motor strategies in elite athletes

David Mann presented video footage tracking the head movements and gaze of Australian cricketers during batting and showed some remarkable differences between the strategies of elite-level batsmen versus less skilled players - in particular, the elite-level batsmen were better able to couple their head movements with the flightpath of the ball, and they were able to predict the ball’s flightpath such that they could watch the ball as it came in contact with the bat, which is something that their less skilled counterparts did not do. David also provided footage from research conducted in football goalkeepers and table tennis players.

Above all else, I was amazed at how technology had come so far as to allow such recording of elite sportspeople’s gaze, without risk of observer effect. With the Australian cricket team’s recent success in the World Cup, I imagine there will be a few people taking a keen interest in these findings.

## The invited symposium on exercise in cancer patients

As someone with a background in clinical trials, this symposium was as relieving as a soak in a nice, warm bath. After 2 days of presentations about studies conducted in ‘a dozen healthy participants’, finally, this symposium offered some nice, large, randomised controlled trials!

Firstly, Karen Steindorf presented results from two clinical trials: investigating the efficacy of a 12-week resistance training programme with regard to relieving cancer-related fatigue in stage 0-III breast cancer patients undergoing adjuvant chemotherapy (*n* = 101 patients) and radiotherapy (*n* = 160), respectively.

Next, Laurien Buffart and Anne May presented results from two large Dutch studies: the Physical Activity during Cancer Treatment study and the Alpe d’HuZes Cancer Rehabilitation study, which together looked at the effects of physical activity and exercise programmes on a number of different outcomes, including fatigue, physical fitness and quality of life in patients with various types of cancer.

Lastly, Morten Quist presented findings from EXHALE: a randomised controlled exercise intervention trial involving more than 200 patients with advanced-stage lung cancer.

## The plenary session entitled, ‘Challenges for the paralympic games: fairness and identity’

Between them, the two presenters – Yves Vanlandewijck and Mike McNamee – considered how the Paralympic Games has evolved from a participation-oriented event for those with disabilities, to an elite event involving, highly trained, (often) full-time, professional athletes using some very high-tech equipment. Yves Vanlandewijck addressed how growing diversity threatens the fairness of the competition; while Mike McNamee addressed how technological innovations such as robotics, prosthetics and exoskeletons challenge some of the core values of the Paralympic movement, namely ‘equality’ and the ability to ‘inspire’.

The case of the able-bodied Romanian ATP tennis player, Simona Halep, who had recently undergone a breast reduction to relieve back pain and, ultimately, improve her game, was presented, with the question raised of whether it is also ethical for paraplegic athletes to amputate their lower limbs (or amputees to reduce the length of their lower limbs) to improve manoeuvrability and performance. . . and, if so, where can the line be drawn? With running blades evolving in such a way that ‘disabled’ runners may soon run faster than their able-bodied counterparts, will we one day see athletes selectively undergoing lower limb amputation in order to improve performance?

This was a truly thought-provoking session.

## The young investigator award winner - Mike Stembridge

Finally, I would like to conclude by recommending to any conference-goer (*just to make the conference that little bit more interesting*): take dibs amongst your friends as to who’s going to win the Young Investigators’ Award. I was delighted when the investigator I was backing, Mike Stembridge, pulled through, with his presentation on left ventricular mechanics at rest and during exercise at high altitude.

In support of previous studies, Mike showed that, in healthy subjects, resting stroke volume is reduced at high altitude compared with sea level. His research went on to show, however, that left ventricular twist, apical cicumferential strain and ejection fraction were all increased with altitude, which he suggested possibly represented a protective mechanism against an even greater reduction in stroke volume. He proposed that the higher resting left ventricular twist and apical circumferential strain reduces one’s functional mechanical reserve, as his subjects’ ability to increase stroke volume with exercise at high altitude was significantly limited.

Mike Stembridge’s clear presentation made some quite complex research seem understandable.

I would like to wish all those attending, a most enjoyable 20th anniversary ECSS meeting in Malmö this year.

